# Editorial: Murine Models of Leukemia and Lymphoma

**DOI:** 10.3389/fonc.2017.00309

**Published:** 2017-12-08

**Authors:** Christine E. Cutucache, Pierluigi Porcu

**Affiliations:** ^1^University of Nebraska Omaha, Omaha, NE, United States; ^2^Sidney Kimmel Cancer Center, Philadelphia, PA, United States

**Keywords:** murine models, leukemia, lymphoma, tumor cell plasticity, cytokines/chemokines

**Editorial on the Research Topic**

**Murine Models of Leukemia and Lymphoma**

Murine models serve as an effective way to mimic the *in vivo* tumor microenvironmental interactions that take place in patients with leukemias and lymphomas. Specifically, leukemias and lymphomas rely heavily on the surrounding stroma and tissue microenvironmental cytokine and chemokine signals to ensure survival and expansion of tumor cells. Finally, leukemic cells migrate thanks to signals from varying regions of the host, furthering the progression and severity of disease. It is therefore impossible to fully understand such a dynamic relationship between tumor cells and their surrounding microenvironment, and the events to transformation in leukemias and lymphomas without an *in vivo*, or murine model. While many models have been established, their strengths and weaknesses must be realized in order to best interpret findings from each model and ultimately apply that to the clinic. All of this knowledge is useful in determining the etiology of the tumor cells, the plasticity of them, their relationship with the surrounding microenvironment, diagnostic and prognostic markers, as well as serve as a way to test novel therapeutic regimens.

## Author Contributions

All authors listed have made a substantial, direct and intellectual contribution to the work, and approved it for publication.

## Conflict of Interest Statement

The authors declare that the research was conducted in the absence of any commercial or financial relationships that could be construed as a potential conflict of interest.

